# Towards human exploration of space: the THESEUS review series on muscle and bone research priorities

**DOI:** 10.1038/s41526-017-0013-0

**Published:** 2017-02-14

**Authors:** Thomas Lang, Jack J.W.A. Van Loon, Susan Bloomfield, Laurence Vico, Angele Chopard, Joern Rittweger, Antonios Kyparos, Dieter Blottner, Ilkka Vuori, Rupert Gerzer, Peter R. Cavanagh

**Affiliations:** 1 Department of Radiology and Biomedical Imaging Box 0946, University of California, CA San Francisco, USA; 2 DESC (Dutch Experiment Support Center) & 3D InnovationLab, Dept. Oral and Maxillofacial Surgery/Oral Pathology, Dept. Oral Cell BiologyAcademic Centre for Dentistry Amsterdam (ACTA), VU University Medical Center, Amsterdam, Netherlands; 3 Department of Health & Kinesiology, Texas A&M University, College Station, TX USA; 4 Laboratoire de Biologie Intégrée du Tissu Osseux, LBTO INSERM U1059, University of St. Etienne, St. Etienne, France; 5 Dynamique musculaire et métabolisme (DMEM) UMR 866 INRA, University of Montpellier 1, Montpellier, France; 6 Institute of Aerospace Medicine, DLR and Department of Pediatric and Adolescent Medicine, University of Cologne, Cologne, Germany; 7 Department of Physical Education and Sports Science, Laboratory of Exercise Physiology and Biochemistry, Aristotle University of Thessaloniki, Thessaloniki, Greece; 8 Charite Hospital, Berline, Germany; 9 UKK Institute for Health Promotion Research, Tampere, Finland; 10 Skoltech University, Moscow, Russia; 11 Department of Orthopedics and Sports Medicine, University of Washington Medical Center, Seattle, WA USA

## Abstract

Without effective countermeasures, the musculoskeletal system is altered by the microgravity environment of long-duration spaceflight, resulting in atrophy of bone and muscle tissue, as well as in deficits in the function of cartilage, tendons, and vertebral disks. While inflight countermeasures implemented on the International Space Station have evidenced reduction of bone and muscle loss on low-Earth orbit missions of several months in length, important knowledge gaps must be addressed in order to develop effective strategies for managing human musculoskeletal health on exploration class missions well beyond Earth orbit. Analog environments, such as bed rest and/or isolation environments, may be employed in conjunction with large sample sizes to understand sex differences in countermeasure effectiveness, as well as interaction of exercise with pharmacologic, nutritional, immune system, sleep and psychological countermeasures. Studies of musculoskeletal biomechanics, involving both human subject and computer simulation studies, are essential to developing strategies to avoid bone fractures or other injuries to connective tissue during exercise and extravehicular activities. Animal models may be employed to understand effects of the space environment that cannot be modeled using human analog studies. These include studies of radiation effects on bone and muscle, unraveling the effects of genetics on bone and muscle loss, and characterizing the process of fracture healing in the mechanically unloaded and immuno-compromised spaceflight environment. In addition to setting the stage for evidence-based management of musculoskeletal health in long-duration space missions, the body of knowledge acquired in the process of addressing this array of scientific problems will lend insight into the understanding of terrestrial health conditions such as age-related osteoporosis and sarcopenia.

## Introduction

The musculoskeletal system is central to work, locomotion, and posture. Maintaining its integrity during long-duration spaceflight is essential to mission completion as well as to astronaut health during and after the mission. One of the principal obstacles facing the design and implementation of long-duration exploration class missions is the fact that, without countermeasures, all components of the musculoskeletal system are altered by the environment of the long-duration mission. These comprise exposure to microgravity, amounts and characteristics of space radiation that differ from those of Earth and even from those of low Earth orbit, as well as changes in diet and light exposure that can impact the metabolism of musculoskeletal tissues. Deleterious changes noted in ground-based models as well as the current evidence base of over 30 years of long-duration missions in low orbit (over 15 years of experience in the International Space Station, ISS) include extensive bone loss, loss of muscle mass and strength, increased risk of kidney stones, vertebral disk alterations, and lower back pain as well as changes to the elasticity of the tendons. In the absence of countermeasures, these changes can be severe, potentially impacting the safety and performance of crew members during extravehicular activities (EVA) in microgravity and partial gravity environments, and putting them at risk for injuries and other health impairments upon return to Earth.

This review summarizes and expands on the discussions that took place in the Bone and Muscle Advisory group of the project entitled “Towards Human Exploration of Space: a European Strategy (THESEUS). The aim of THESEUS was to recommend a set of research priorities to the European Space Agency (ESA) for the preservation of health and performance of ESA astronauts, with the goal of suggesting approaches that would build on the technical and economic strengths of the European Community. The goal of this review is to describe the base of knowledge accumulated thus far regarding the changes to the musculoskeletal system in long-duration spaceflight and the efficacy of countermeasure approaches for preventing or at least reducing the impact of those changes. We will review evidence from ground-based and space-based studies in both human and animal models, and recommend priorities for further studies required to understand and mitigate the risks to the musculoskeletal system associated with very long duration missions beyond low-Earth orbit, such as to Mars or asteroids.

## Bone and muscle loss: evaluations in spaceflight

### Early findings of bone loss in long-duration missions

Pre-flight and post-flight studies carried out on the Russian MIR and early ISS missions, studies of astronauts performed pre-flight and postflight have documented the magnitude and regional variation of bone loss. In a study of 26 MIR cosmonauts undergoing bone mineral density (BMD) measurements with dual x-ray absorptiometry (DXA) before and after spaceflights lasting 6 months on average, LeBlanc *et al*. reported losses of BMD averaging around 1% per month of spaceflight at the spine and femoral neck and 1.5% per month at the trochanter.^1^ Thus, in roughly 1 month of spaceflight, these subjects incurred a loss of BMD typically observed over a year in a postmenopausal woman. In a study of members of the first eight ISS crews, Lang *et al*. employed quantitative computed tomography (QCT) to image changes in hip and spine volumetric BMD (vBMD) over flights of similar lengths.^2^ Compared to DXA, which measures the integral bone compartment, combining changes in both the trabecular and cortical bone, QCT allows for separate depiction of the bone compartments. Similar to the earlier MIR study, Lang *et al*. found that ISS crew lost 1–1.5% per month integral vBMD at the hip and spine, but QCT measurements showed that the cortical and trabecular vBMD changed at different rates, with trabecular bone losses at the hip ranging from 2–2.7% per month, with losses of cortical BMD less than 1% per month and for some proximal femoral subregions, not statistically significant.^2^ While hip cortical vBMD changed relatively little over the flight, measures of cortical bone mass and volume changed at the rate of 1% per month, consistent with the integral measurements, and indicating that bone was lost by decreased cortical thickness rather than loss of density. The severe losses of bone observed at the proximal femur were not observed in the spine. At the spine, this study reported a low rate of trabecular bone loss (<1% per month), but losses in the integral and cortical compartments, as well as the posterior processes, which were similar or slightly larger than was reported in Leblanc *et al.* ^1^ In order to directly assess the impact of spaceflight on the strength of the hip, Keyak *et al*. analyzed the QCT data from the ISS study using finite element modeling (FEM).^3^ computed tomography (CT)-based FEM depicts bone strength by using imaging to construct models incorporating subject-specific maps of material properties and bone geometry, subjecting those models to simulated loading forces representing lateral falls or single-legged stance.^4–6^ By estimating the mechanical load required for the hip to fracture, CT-FEM provides a more clinical relevant measure of skeletal integrity. In the FEM studies, Keyak observed that astronauts lost 2.4–2.7% of their hip strength per month of spaceflight, comparable to the trabecular losses and larger than those observed for BMD.^3^ The variability of loss was very high, with one of 13 subjects experiencing a strength loss of 50%, more than the lifetime loss associated with aging over the course of a 6-month mission. In contrast, other subjects experienced no detectable strength loss. Other investigators have examined changes in vBMD in the peripheral skeleton using smaller peripheral CT scanners designed to scan the distal radius and tiba. Vico *et al*. observed in a study of 11 cosmonauts with 6-month MIR flights, a mean loss of trabecular vBMD from the distal tibia of roughly 5%, with a 1.7% mean loss of cortical bone, similar to the proximal femur study.^7^ They also observed, in keeping with the earlier MIR results, minimal loss at the distal radius, indicating that the greatest changes are observed in the load bearing skeleton.

Studies following up bone density measurements in the years after spaceflight show varying patterns of recovery, depending on the measurement approach. Sibonga *et al*. followed DXA BMD measurements in astronauts after completion of missions averaging 6 months in length.^8^ They found that recovery of DXA bone density fit an exponential pattern, was incomplete 1 year from flight, but approached baseline levels asymptotically over the next 2 years. Studies employing volumetric QCT show that, for the proximal femur, recovery after spaceflight is a process involving differential changes in cortical and trabecular bone density, and in size.^9, 10^ A second study by Lang *et al*. included measurements taken 1 year after the completion of spaceflight.^10^ This study found that although integral BMD and bone mineral content were recovered 1 year after spaceflight, trabecular bone and integral volumetric bone density did not recover fully. In fact, during the year after flight, the size of the proximal femur increased as measured by proximal femur volumes and femoral neck cross sectional area. These bone size measures were significantly larger at 1 year than pre-flight. The presence of larger bone size, thinner cortices and lower trabecular bone density indicated that the combination of spaceflight and post-flight recovery induced irreversible changes in bone, with unknown consequences for fracture at more advanced age. A subset of these subjects were followed by Carpenter *et al*. for 2–4 years after completion of their missions.^9^ They observed that while the recovery of proximal femoral trabecular bone tended to drop off or reverse, cortical vBMD continued to increase, and thus, modest increases in cortical bone density may be a key factor in the asymptotic recovery of DXA BMD.

### Countermeasure studies

Countermeasures to bone loss have involved use of exercise to replace, or at least partially replace mechanical loads sustained in 1 g. Initial countermeasures available on MIR involved a treadmill system and exercise bicycle. Cosmonauts also made use of a “penguin suit”, which tended to force the body into a fetal position and in which continuous muscle forces were required to maintain the torso in an extended position.^11^ On the ISS, early countermeasures, up to 2004, included an Interim Resistance Exercise Device (iRED),^12^ as well as a treadmill (in which bungee cords were used to tension crew to the device) and exercise bicycle. Despite the presence of resistance and aerobic exercise countermeasures on both the MIR and early ISS missions, early crews still showed extensive bone loss.^2^ In 2004, NASA installed the advanced resistance exercise device (aRED),^13^ which had higher loads aimed at increasing forces on bone, particularly the spine and proximal femur. Initial results from crew members who exercised on the aRED were encouraging. In 2012, Smith *et al*. carried out a study on five ISS astronauts (mission length 48–215 days) who exercised on the aRED, comparing energy intake, vitamin D levels, bone resorption markers and BMD measurements to 8 previous ISS crew who had exercised on the iRED device.^14^ Compared to crews who exercised on the earlier iRED device, these subjects had higher energy intake (90% of World Health Organization recommendations compared to 70–80% for the initial crews) and higher Vitamin D levels due to an increase in supplementation from 400 to 800 International Units per day. The skeletal response was monitored through observation of bone metabolism markers throughout the missions and pre-flight and post-flight measurements of BMD. Markers of bone metabolism, both formation and resorption, increased in both iRED and aRED exercisers, but data suggested that aRED exercise attenuated the resorption increase relative to iRED. The iRED, but not the aRED exercisers, showed a decrease in BMD through the flight. Postflight BMD in the five crewmembers, who exercised on aRED, was not statistically different from preflight BMD. Compared to iRED exercisers, aRED exercisers returned with higher percentage lean mass and lower percentage fat mass. A subsequent study examined whether adding anti-resorptive treatment to aRED exercise was beneficial in reducing bone loss. Using indices of bone density and strength derived from QCT and bone densitometry by DXA, Leblanc *et al*. compared a group of astronauts combining aRED exercise with the anti-resorptive medication alendronate (*n* = 7) to untreated subjects exercising on the aRED (*n* = 11), with both groups compared to a control group of iRED exercisers (*n* = 18).^15^ The combination of an anti-resorptive drug and aRED exercise attenuated all indices of bone loss by DXA and QCT. Although their levels of bone loss at hip and spine tended to be lower than those on the iRED, untreated subjects treated on the aRED experienced significant decreases from baseline in DXA BMD measures of the femoral neck and total hip, and these losses were statistically significant when compared to the treated aRED exercisers as well. Studies of bone biochemistry of exercising subjects, both iRED and aRED, show increased levels of both bone resorption and formation during spaceflight.^16^ aRED exercise tended to increase the level of bone formation markers without appreciably reducing the elevated resorption markers, which were attenuated only when anti-resorptive treatment is added to exercise. Thus, high-intensity resistance exercise shows strong evidence for substantially reducing the rate of bone loss in long-duration spaceflight as well as maintaining lean mass and physical condition. Part of the effect may come from the fact that astronauts are told to eat more in order to achieve the nutrient intake levels required for their physical activity, with both the increased loading of the musculoskeletal system, and the increased nutritional intake contributing to a preservation of bone and muscle.^14^ However, while exercise appears to improve the bone turnover balance by increasing bone formation markers, there is still significant loss at a lower level, which is only attenuated by use of an anti-resorptive medication.

### Findings of muscle atrophy on Shuttle and on early long-duration missions

Data from spaceflight studies indicate severe loss of muscle mass/volume and strength, despite the use of exercise countermeasures, even in short-duration missions. Following an 8-day Space Shuttle mission, LeBlanc *et al*. observed significant losses in the soleus-gastrocnemius (−6%), hamstrings (−8%), quadriceps (−6%), intrinsic back muscles (−10%), and the anterior calf muscles (−4%).^17^ Similarly, Akima *et al*. observed losses in the quadriceps (−6 to −15%), hamstrings (−6 to −14%), and ankle plantarflexors (−9 to −16%) in three crewmembers on missions ranging from 9 to 16 days.^18^ Biopsy of the vastus lateralis (VL) muscle following an 11-day flight demonstrated that muscle atrophy was apparent at the level of the myofiber, ranging from 16 to 36%.^19^ Despite exercise countermeasures, initial data from long duration flights indicated extensive losses in muscle volume. Following missions of 16 to 28 weeks, quadriceps (−12%), hamstrings (−16%), intrinsic back (−20%), gastrocnemius (−24%), soleus (−20%), and anterior leg (−16%) muscle volume were reduced.^17^ As expected, reduced muscle strength was concurrent with the losses in muscle mass, but the magnitude of the changes in muscle strength appeared to be somewhat greater. This result was consistent with much shorter Space Shuttle missions, for example, where knee extensor strength was reduced by 12% and trunk flexor strength declined by 23%.^20^ In general, it is accepted that the majority of the losses occur in the trunk and the lower body, experienced in those muscle groups which are active in normal 1 g posture and ambulation. Upper body strength changes are less dramatic than those observed in the lower body. LeBlanc *et al*. have suggested that after 4 months of microgravity exposure, the muscle mass reaches a new steady-state, or baseline, condition.^17^ The hypothesis that microgravity causes a fundamental alteration in motor control has also been suggested by Antonutto *et al*.^21^ They observed two legged muscle power to decline considerably more than could be explained by the loss in muscle mass. Additionally, the loss of explosive leg power was associated with a substantial reduction in the EMG activity of the rectus femoris, VL, and vastus medialis muscles.^22^ These authors concluded that microgravity induced a basic change in motor control and coordination such that motor activation of extensor muscles was reduced. Initial studies on board the ISS found that onboard exercise countermeasures were insufficient to prevent muscle atrophy. Treadmill countermeasure exercise on the ISS has been shown to elicit less force under the feet than similar exercise on Earth, suggesting that restraining loads from the subject load devices are inadequate.^23^ Fitts *et al*.^24^ and Trappe *et al*.^25^ found that exercise countermeasures, including resistance exercises on the iRED and treadmill training did not prevent loss of muscle strength and endurance during these early missions.

### Findings on long-duration missions

English *et al*. recently reported findings from 37 ISS crew members studied in expeditions 1–25 (average flight length 163 days).^26^ In Expeditions 1–17, crews exercised on the iRED device, and in Expeditions 18–25 on the aRED device. Isokinetic knee, ankle and trunk extension, and flexion strength were measured at pre-flight, and 5, 15 and 30 days postflight. Significant strength reductions of isokinetic strength of 8–17% were observed across the whole study. On average, aRED exercisers showed a trend towards reduced strength loss compared to iRED exercisers, but the difference in loss rates between machines was not statistically significant. Even with aRED exercise, loss across isokinetic lower body strength measures was statistically significant, ranging from −4.3 to −14.5%. Strength loss tended to be larger in women than in men, but this trend was not statistically significant. Comparing between postflight timepoints, there was a trend for recovery of strength loss, with decrements of 8.5–0.8% observed at 30 days post flight.

### Knowledge gaps

Studies on the ISS have shown that heavy resistance exercise can significantly reduce bone loss and that treatment with anti-resorptive medications (alendronate) combined with heavy resistance exercise can prevent bone loss. Although this work has demonstrated the value of these approaches, considerable additional research is required. More information is required concerning the efficacy of intermittent timing of exercise bouts, on age and sex differences in exercise countermeasure efficacy as well as the efficacy of exercise for protecting other physiologic systems, such as the immune system and brain. There is a need to investigate new frontline therapies against bone loss, including novel anti-resorptive as well as anabolic agents. Finally, there is a need for further bed rest data on combination countermeasure approaches, such as timed nutritional-exercise countermeasures, combined exercise and anti-resorptive treatment, and in the use of anti-oxidants to combat both radiation and disuse.

### Recommendations for future study

While initial studies have hinted at sex differences in bone^27^ and skeletal muscle response ref. 26 to exercise in spaceflight, further analyses with larger sample sizes will be required to fully elucidate these differences. Additionally, the influence of age in the response to exercise countermeasure efficacy should be evaluated. Attention should also be given to testing exercise approaches that combine resistance exercises with balance and proprioception. Such studies could take advantage of novel analog environments such as :envihab,^28^ a facility operated by the German Aerospace Center, which combines aspects of disuse and isolation, with varying atmospheric pressure and a short arm centrifuge. This type of facility should be utilized to determine the protective effect of exercise on brain and immune function when exercise is carried out in isolated environments, while varying other conditions such as intermittent gravity level and other environmental conditions. Studies should be performed that combine nutritional and pharmacologic approaches with exercise to determine if there are synergistic effects. Future facilities such as the large Human Hypergravity Habitat, H3, might be applied to explore chronic applications of hypergravity.^29^

## Bone and muscle loss countermeasures: bed rest studies

Ground-based analogs such as bed rest studies are widely employed as tools to simulate long-duration missions. These studies are required to overcome the main drawbacks of space studies, which are low sample sizes and uncontrolled experimental conditions. These studies are highly translational in that they can be used to model long-term spaceflight-related disuse without the confounding effects of comorbidities present in patient populations. In studies of bone, bed rest models reprise many of the characteristics of spaceflight, including deterioration in bone mass, density and architecture, increase in excreted calcium and changes in biochemical measures of bone turnover. Earlier investigations demonstrated the ability of countermeasure approaches to attenuate disuse bone loss. These include high intensity resistance exercise^13, 30, 31^ resistive vibration^32–37^ as well as nutritional ref. 38 and pharmacologic interventions (alendronate and pamidronate).^39–41^ As with bone, bed rest studies of skeletal muscle loss to a large extent emulate effects of spaceflight on muscle loss, including loss of muscle mass and strength,^1, 42–46^ with changes starting to occur in the frame of days to weeks, compared to months for detectable bone loss.^47^ These effects include fiber type transformations from Type I to Type II and pronounced losses of fiber mass, power and force.^24, 48^ With respect to countermeasure development, a recent study has shown that it is possible to prevent muscle and cardiovascular deconditioning over 14 days of bed rest through optimizing combinations of aerobic and resistance exercise,^49^ while other studies have investigated the efficacy of amino acid supplementation in reducing bed rest-induced muscle deconditioning.^50, 51^

Bed rest studies are particularly useful as testbed for new assays and countermeasure approaches prior to their adaptation for spaceflight studies or routine use. Bed rest studies have allowed for study of more advanced biomarkers of bone and skeletal muscle changes prior to their use in the spaceflight environment.^32, 48, 52–54^ Other studies of bed rest have included reports of the immunoprotective effects of exercise countermeasures,^55^ effects of exercise on molecular biomarkers associated with the Wnt signaling pathway,^56^ as well as evaluation of effects of exercise on gene expression in skeletal muscle during bed rest.^57–61^ A 2009 study investigated the impact of low amplitude high frequency vibration on the musculoskeletal system, identifying an effect on the intervertebral disk, but with no attenuation of bone loss.^62^ Studies by the Berlin Group and ESA have investigated the technique of resistive vibration exercise, which has been found to attenuate bone loss,^37, 63^ but has not yet been evaluated on the ISS. Other studies have focused on exercise as a countermeasure for alterations of microgravity induced changes in bone biochemistry and renal stone risk.^53, 54, 64–66^

### Knowledge gaps

Exercise protocols have not yet been optimized for time efficiency and synergies with other countermeasures, including nutrition and pharmacologic interventions. Such investigations would benefit from a more refined understanding of the mechanisms of countermeasure actions, which could be obtained by further studies using Omics approaches and transgenic mouse models. Bed rest studies are also important tools for understanding the impact of exercise countermeasures on other physiologic systems related to spaceflight. There is considerable evidence for positive effects of exercise on brain plasticity and function^67^ and immune function^68^ in Earth populations, and a recent study has demonstrated the impact of exercise on immune biochemical factors in bed rest,^55^ there is little knowledge available regarding these relationships in the spaceflight setting. With respect to pharmaceutical treatment, there is lack of information on dose-response relationships of drug approaches to combat bone loss (e.g., bisphosphonates) and on synergies between exercise and anti-resorptive treatment.

### Recommendations for future study

Continued research with larger sample sizes than in the past, and controlled conditions will be required to optimize exercise protocols for efficacy and time efficiency. Achieving this goal will require further studies to elaborate the mechanisms of countermeasure effectiveness in bone and muscle. Exercise protocols require significant investment in crew time, as well as investments in the design and placement of the equipment. In order to maximize the return on this investment, we believe that further bed rest studies are warranted to evaluate the efficacy of exercise for protecting other physiologic systems from the effects of spaceflight. These include brain (behavior/cognition/performance), the neuromuscular system and the immune systems. For pharmacologic interventions, dose response studies are required, and new antiresorptive or anabolic pharmaceuticals, which may provide fewer side effects than bisphosphonates should eventually be tested. There is a need to examine how exercise and drug treatment can operate synergistically in reducing muscle and bone atrophy. Finally, it is important to note that although it exhibits patterns of muscle loss and bone loss similar to spaceflight, bed rest does not fully simulate the conditions of spaceflight as they affect human physiology, including radiation exposure, isolation, fluid shifts, exposure to partial gravity and the space radiation environment^47^ as well as the vestibular system. While space radiation effects cannot be simulated in a human ground analog, other effects, such as isolation, oxygen reduction and pressure decrease, and exposure to artificial gravity may be investigated using facilities such as :envihab or other ground-based analogs (please see above).

## Bone and muscle loss countermeasures: biomechanical forces during long-duration spaceflight activities

The musculoskeletal systems of astronauts experience a range of applied loads across multiple phases of long-duration spaceflight. During periods of exposure to microgravity, loads are imposed on bone and muscle during onboard exercise activities, ranging from resistance exercise to treadmill use, to cycle ergometry. During EVA undertaken in partial gravity environments such as the Moon or Mars, there are variable loads associated with the types of physical activity, such as bending, squatting, shoveling etc. In addition to the type of physical activity, further individual variation is associated with spacesuit design. Development of computational^69, 70^ and experimental techniques^71, 72^ to respectively predict and measure the loads exerted on bone, muscle and cartilage across the range of physical activities, and spacesuit designs is essential both to understanding the potential protective effect of EVA activities on the musculoskeletal system as well as to identify variants of exercise and EVA activities that may result in injury. To this end, the development of vertical treadmill systems^73^ and lower body negative pressure systems^74^ has been instrumental to understanding how variations of load under partial gravity may provide different degrees of osteoprotection and protection against muscle loss.

### Knowledge gaps

Insight is needed regarding how altered patterns of locomotion in partial g scenarios may lead to increased risk of injury. This estimate of injury risk will depend on focused study of the kinematics and kinetics of simulated construction, exploration, and EVA activities, which are not well known. To this end, research should focus on comprehensive simulations of work tasks in partial gravity that should be conducted to aid in the assessment of fitness for duty after prolonged exposure to partial gravity and to assess the risk of injury.

### Recommendations for future study

To better characterize the spectrum of physical activities during actual and simulated EVA, Acceleration Monitoring Units should be added to spacesuits and space exercise systems. In addition, simulations should exploit the availability of ground-based suspension models, which offer considerable possibility for simulation of locomotor and other activities (such as jumping, work tasks, etc.). To better understand the implication of EVA activities for problems such as bone loss, musculoskeletal modeling techniques ought to be refined to generate validated loading estimates of structures (such as the proximal femur) that are at risk for bone loss. These simulations should incorporate altered gait patterns in partial gravity that might lead to injury, in particular those associated with work tasks. The information to be gained from these studies will be critical for optimizing design of spacesuits and footwear for EVA.

## Musculoskeletal injury in long-duration spaceflight

Current information suggests that healing of both hard and soft tissues is delayed in microgravity. At the molecular/cellular level, fibroblasts, muscle cells and extracellular matrix are sensitive to alterations in gravitational forces.^75–77^ Limited data from space-flown animals have shown that the repair process of load-bearing bone may be hindered during acute exposure to microgravity.^78^ In addition to fractures, ligament tears, and muscle tears, back pain due to expansion and/or degeneration of the vertebral disk is another important source of musculoskeletal injury.^79^ Information from Earth-based studies of fracture healing in animal models and back pain in bed rest studies should inform experiments on the healing of ligaments and tendons, as well as disk degeneration, since unloading is a common form of treatment on Earth.

### Bone fracture

Ground-based animal models simulating microgravity, currently represent the only mechanisms for studying the potential effects of space flight on bone fracture healing. These models allow for control of fracture mechanics, reloading condition and environmental variables, providing important mechanistic information. While most studies have employed the rodent-based hindlimb unloading (HLU) model developed by Morey-Holton *et al*.,^80^ other studies have employed larger animal models,^81^ in which unilateral limb suspension was employed to evaluate the effect of unloading vs. loading on the rate and quality of fracture healing. Evidence from animal models supports the idea that chronic disuse associated with the spaceflight environment result in delayed fracture healing and compromise in the quality of the bone after healing. Rodent HLU studies, in particular, have yielded interesting insights, such as skeletal site-specific variations in the impact of unloading on fracture healing of tibia and femur.^82^ These studies have provided insights into mechanisms by which HLU affects fracture healing through reduction of angiogenesis and resulting vascularity of the fracture site.^83, 84^ Other studies have shown that the unloading environment compromises the regenerative capacity of bone tissue by inhibiting differentiation of mesenchymal cell precursors into osteoblasts and promoting their differentiation in adipocytes.^85^

### Ligaments and tendons

There is evidence that collagen fiber orientation in tendons may be altered by microgravity.^86, 87^ If verified, this may have important implications for recovery of soft tissues from long-duration spaceflight. Additionally, there are data suggesting that unloading of the rat hind-limbs for 3 or 7 weeks inhibits dense fibrous connective tissue wound healing processes, suggesting that some minimum of mechanical loading is required for effective connective tissue repair.^88^ Further information is available from human bed rest studies where an aggressive exercise strategy designed to prevent bone and muscle loss, high rates of soft tissue injury were observed.^30^ A literature review of shoulder injuries attributed to resistance training^89^ has indicated that predictors of soft tissue injury remain ill-defined and cited the need for further research in this area.

### Back pain

For returning astronauts, the risk of lumbar and cervical IV disk herniation is almost a factor of 3 higher than for the general population; the prevalence of back pain in astronauts is approximately twice that of the general population.^79^ Of 321 crew members from Shuttle, MIR and ISS missions described in a 2010 study, almost 10% were found to have herniated disks after spaceflight.^90^ The etiology of disk herniation and lower back pain in astronauts is complex and may include several factors, such as increased hydration and swelling of the disk during microgravity, as well as deterioration of the muscles of the neck and lower back, which can impact the ability of the vertebral column to bear axial load during spaceflight.^79^ In patients with lower back pain, cross sectional area of the paraspinal muscles is reduced by 10%. In clinical back pain, this decrement in muscle area can be accompanied by imbalances in loss between the back muscles, as muscle atrophy can vary side to side and between muscle groups. A recent study evaluated changes in the back musculature in a 60-day bed rest using MRI to characterize volumes of the spinal muscle groups.^91^ In a group of non-exercising controls, they observed differential rates of loss in the multifidus, erector spinae and psoas, which translated to changes in peak trunk flexion and extension torque. In an exercise group, a combination of lower body negative pressure and flywheel exercise attenuated muscle losses by over 50%. Evidence indicates that recovery from back muscle atrophy is a prolonged process, with recovery of muscle volume, and strength taking on the order of weeks and the recovery of overall function requiring months.^79^

#### Knowledge gaps

Rotator cuff injuries and other joint injuries^92^ are common in astronauts during training and flight,^93^ and more information is required on the etiology of these injuries. There is a lack of information in general about the effects of altered gravity on ligaments and tendons. These tissues have been somewhat ignored in favor of studies of bone and muscle. The enthesis (i.e., mechanically stressed connective tissue structures of the muscle–bone interface) is a common source of problems in sports medicine.^94^ However, information on the response of the enthesis to altered gravity is absent from the literature. What is the effect of various exercise activities on tendons and ligaments? What are the predictors of injury to tendons and ligaments? Medical management procedures for inflight fractures need to be elucidated and standardized. The mechanisms of spaceflight-related back pain need to be elucidated.

#### Recommendations for future study

Expanded investigation is required in the areas of ligament and tendon in spaceflight. Ideally, this could lead to an identification of the key risk factors for injury. Little is known about adaptations at the enthesis during space flight and animal studies that address this issue are needed. It would be beneficial to consider the effects of specific countermeasure exercises in key soft tissue structures that are at risk of injury. Research to safeguard the integrity of the shoulder in space is urgently needed. With respect to simulations of fracture healing, rodent experiments are warranted in which fractures are induced during flight, and in which the live animal is returned for imaging and histological studies of healing impairment. Such experiments could be complemented by the induction of fractures while in space. With respect to back pain, animal models of conditions leading to back pain need to be developed in order to understand etiologic factors and to test interventions. In human studies, there is a need to do more correlative studies between activity, exercise and spine morphology, etc. to gain insight into predictors of back pain. There is also a need for detailed studies of the various tissue compartments (muscles, disks, ligaments, etc.) whose changes are associated with back pain. Finally, the study of musculoskeletal injury presupposes the question of how such injuries would be managed in long duration spaceflight scenarios. Since ultrasound is likely to be the only imaging modality available, research needs to be focused on the diagnostic and therapeutic use of ultrasonic techniques.

## Scientific/mechanistic aspects: facilities for animal studies

Animal research represents the principal approach to obtain mechanistic information regarding some of the most important biomedical problems facing long-duration spaceflight. These studies offer the advantage of large sample sizes, the ability in mouse studies to use genetic manipulation to experimentally evaluate the effect of genetic factors on the response to the spaceflight environment, the ability to evaluate tissue effects of the spaceflight environment in situ at a microscopic level and compare to the predictions of computational bone adaptation models, and the capability to manipulate radiation exposures to study the detailed effects of the space radiation environment. Animal research into microgravity effects have primarily been based on the hindlimb-unloading model, with more recent introduction of variants simulating partial gravity environments. Hindlimb unloading, however, does not fully correspond to microgravity, and studies that combine HLU with other spaceflight-related factors such as radiation exposure are experimentally complicated. The limitations of HLU are now being partially addressed by facilities being flown on the ISS. The Italian Space Agency, developed the Mouse Drawer System (MDS) that flew for 91 days on ISS.^95^ A mouse facility, BOS, was also launched on board the Russion Bion-M1 mission.^96^ The Animal Enclosure Module, housed at NASA Ames Research Center, is a system modified from the Shuttle era in which up to 8 mice can be transported to the ISS aboard a SpaceX Dragon cargo spacecraft and then transferred to one or more sealed habitat modules. Upon experiment completion, animals are returned to Earth with the Dragon and then sent for postflight measurements and processing. The Mouse Habitat Unit, housed aboard the Japanese Kibo Module, is equipped with 12 environmentally controlled mouse cages. The system, which like the Animal Enclosure Module, allows for live return of animals, is equipped with a centrifuge facility allowing for exposure of flown animals to partial and full gravity environments.

### Knowledge gaps and future studies

Evaluating the impact of partial gravity on the musculoskeletal system in animal models is a critical step for understanding the musculoskeletal impact of long sojourns in settings such as the moon or mars, where gravitational forces are respectively, 0.17 and 0.38 g. Moreover, understanding of the minimum level to which gravitational loading must be replaced is also critical to the design of artificial gravity systems. Thus, these studies should continue with high priority. Additionally, non-weight-bearing, non-mammalian systems such as Medaka and Zebrafish appear to demonstrate changes to bone cell activities associated with changes in gravity.^97, 98^ Thus, evaluating these non-mammalian models in comparison to traditional rodent models could be applied to better elucidate effects of altered gravity on bone and muscle.

## Scientific/mechanistic aspects: genetic predisposition

Genome-wide association studies have identified specific genes associated with both bone density incident bone fracture in epidemiologic studies.^99, 100^ Animal models show differences in muscle and bone phenotypes based on genetic variation, and animal experiments have revealed genetic associations of loss of bone microstructure and strength under HLU.^101, 102^

### Knowledge gaps

At present, there is a dearth of integrated knowledge in this area, which can be directly applied at the human level to such activities as crew member selection for flight based on genetic profiling. Experimentation on the wide variety of genetically modified animals has, to date, been confined to simulated microgravity in 1 g conditions.

### Recommendations for future study

Detailed family histories of musculoskeletal diseases, including the incidence of osteoporosis and fractures, should be collected on all crew members and these data should be compared with pre-flight and post-flight measures of musculoskeletal status. Better animal facilities, including an inflight centrifuge for generating inflight controls or partial gravity profiles, are urgently needed on the ISS and animal studies with genetically modified rodents should focus on those animals that have shown resistance to the loss of musculoskeletal during 1 g-based models of unloading.

## Mechanistic aspects: sex differences

Sex differences in the musculoskeletal complications of spaceflight may require the development of sex-specific strategies for countermeasures, as well as provide insight into sex-specific complications of aging. Although a study of ISS astronauts (33 men, 9 women) concluded that women may, in general, have better preserved their musculoskeletal tissues during the missions compared to men,^27^ other studies showed that male and female crew had similar rates of whole-body and regional bone loss.^66, 103, 104^ Thus, studies of sex-specific countermeasure responses, obtained with larger sample sizes, are merited, despite the extra costs of including sex as a variable. However, there are many unanswered questions regarding these observations including medication use (including estrogen), pre-flight BMDs, exercise countermeasure profiles etc. While bed rest studies involving women have shed new light on the response of women to unloading and inactivity,^48, 52, 105, 106^ there is still much to be learned.

### Knowledge gaps

It is critically important to identify the mechanisms for the different patterns of musculoskeletal deconditioning that have been identified in women compared to men. To date, most women on long-duration missions have suppressed their menstrual cycles through pharmacological intervention. The effect of such a suppression over a prolonged period such as a Mars mission is unknown. Similarly, the effects of space radiation in the presence of estrogen are unknown and also require further investigation, perhaps initially in animal model studies.

### Recommendations for future study

There is an urgent need for bed rest studies in which men and women are exposed to exactly the same conditions and countermeasures during the period of confinement. Similarly, side-by-side animal studies using male and female animals need to be conducted in order to explore sex-based differences at the level of cellular and molecular kinetics. The response of women to simulated and actual unloading in relation to their menstrual cycles needs to be studied. The design of exercise-countermeasure equipment must take the differing body dimensions of men and women crew members into account so that the largest and smallest crew members can exercise over a full range of loads and motions without undue hardship.

## Mechanistic aspects: Radiation studies

The closest clinical analogy to space radiation is clinical radiation therapy, which involves entirely different doses and types of radiation. However, information gained from mechanistic studies may shed light on potential cellular and molecular pathways by which bone and muscle may be affected in the clinical radiation therapy setting. Animal studies have shown that exposure to heavy ions that simulated galactic sources of radiation induced rapid transient osteoclastic bone resorption.^107^ Other studies of cultured osteoblasts have shown deleterious effects of radiation on osteoblast proliferation and function ref. 108 as well as on bone lining ref. 109 and hematopoietic cells that are related to bone function.^110^ The effects of drug and nutritional interventions on musculoskeletal status have also been studied refs. 111, 112 in the simulated space environment of HLU and radiation exposure.

### Knowledge gaps

Definition of the risk to musculoskeletal tissues posed by the space radiation environment requires continuation of current animal studies establishing cellular and molecular pathways for bone loss, and the extension of these studies to examine effects on muscle.

### Recommendations for future study

Mechanistic studies should build on intital studies involving different radiation types, and should examine effects in the context of microgravity as simulated by the HLU model.^113–117^ These mechanistic studies should also define the ability of radiation-damaged musculoskeletal tissues to recover function. Future animal studies should begin to assess the effect of countermeasure approaches to radiation induced bone and muscle loss, including anti-resorptive therapies for bone, and anti-oxidants for bone and muscle.

## Conclusions

### Countermeasures for bone and muscle loss

The development of effective, time-efficient, comprehensive musculoskeletal countermeasure approaches are essential to mission success in long-duration spaceflight, since integrity of musculoskeletal tissues and their function is indispensable to optimal performance and injury avoidance on partial gravity surfaces. Compared to initial studies demonstrating extensive losses of bone density and strength in early long duration spaceflight and bed rest studies, countermeasure studies carried out initially in bed rest, and later in long-duration space flight have shown that high intensity lower body resistance exercise, especially when coupled with anti-resorptive treatment, can attenuate or prevent bone loss. While the capability for high-intensity resistance training is available on the ISS, such capability might not exist on long-duration missions beyond low Earth orbit. Thus, an emphasis on optimizing combinations of resistance and aerobic exercise, pharmacologic treatment and nutritional approaches is warranted. Further, it is important to better understand the impact of exercise on other physiologic systems, such as the immune system and brain, as well as to examine biomechanical aspects of exercise to reduce risk of injury.

### Biomechanics

Biomechanical issues are important for understanding a number of aspects of long-duration spaceflight. Locomotion during EVA on a surface with altered gravity has received some attention in the past but much remains to be defined, particularly for locomotion on Mars where no empirical evidence is available and few simulations have been conducted. Knowledge of typical locomotor patterns is also important for the design of habitats where increased vertical movement during gait and enhanced vertical jumping ability will require additional headroom. Biomechanical insight into typical exploration work tasks is important for task scheduling, suit design, and injury prevention.

### Musculoskeletal injury

Injury to musculoskeletal tissues during space flight has the potential to be mission-critical. Many situations can be envisaged in which crew members will be unable to carry out important, and in some cases life preserving, maneuvers because of musculoskeletal injury. Bone fracture has, appropriately, been the focus of most prior spaceflight injury research, but even in this area much remains to be learned and contradictory findings need to be explained. However, injury to ligaments and tendons—which are so common in terrestrial workplace and sports settings—are likely to occur during long-duration spaceflight in the future and very little research to date has addressed mechanisms for healing in altered gravity. The potential for differential rates of recovery of muscle, fascia, tendons, ligaments, and bones to injury on return to Earth presents significant challenges to post-flight rehabilitation. Back pain has proven to be one of the most common complaints of returning crew members to date and it is critical that a better understanding of its etiology and treatment be achieved.

### Ground-based animal studies

Expanded investment in ground-based animal studies is required to generate mechanistic information to serve as the basis for development of countermeasures to the microgravity and radiation environment of space. The ability to model radiation and fracture healing effects are two key aspects of animal studies that address major spaceflight risks but which cannot be ethically studied in humans. Use of facilities such as the MDS, Bion facility, the Animal Enclosure Module, the Mouse Habitat unit and habitats for aquatic models, coupled with appropriately designed transgenic animal studies, will be instrumental to obtaining a mechanistic understanding of the impact of the space environment on the muscloskeletal system.

### Sex differences

Much remains unknown about the effect of sex in the response to altered gravity. While this is not surprising given that women crew members have represented less than 20% of all fliers to date, there is a compelling need to address this sex-based disparity in information and research. While a volume of data will become available as more women fly, investment in bed rest studies is warranted to better understand the role of sex as a factor in the effects of spaceflight.

### Radiation

The environment of deep space involves exposure to galactic and solar radiations that are largely deflected by the Earth’s magnetic field. Recent studies have indicated that exposure to this radiation may play an important role in bone loss, and the effect on muscle is not yet known. Thus, a mechanistic understanding of radiation-induced bone loss, based on realistic simulations of the space environment, is essential to understanding the magnitude of the risks involved and to development of countermeasures. While there is some evidence that radiation disrupts muscle tissue at the fiber level, the state of knowledge is much less advanced than it is for bone, and development of the field is of high priority. This work will be highly interdisciplinary and is likely to produce knowledge that will be of value to the study of other physiologic systems.
